# Antimicrobial Resistance in Bacteria from Livestock and Companion Animals

**DOI:** 10.3201/eid2512.191193

**Published:** 2019-12

**Authors:** Laurel Redding

**Affiliations:** University of Pennsylvania, Kennett Square, Pennsylvania, USA

**Keywords:** antimicrobial resistance, livestock, companion animals, bacteria

In this era of “superbugs” and rising antimicrobial resistance, *Antimicrobial Resistance in Bacteria from Livestock and Companion Animals* (Figure) is a valuable resource to better understand the contribution of animal-derived pathogens to this growing public health crisis. The use of antimicrobial drugs in animal populations is not without controversy; the underlying concern, of course, is that antimicrobial use in animals results in illness and death in humans. This text does not seek to specifically condemn or exonerate. Instead, it provides a comprehensive account of a very complicated topic, delving into the nuances needed to understand the what, where, when, and why of antimicrobial resistance in companion animals and livestock.

**Figure:**
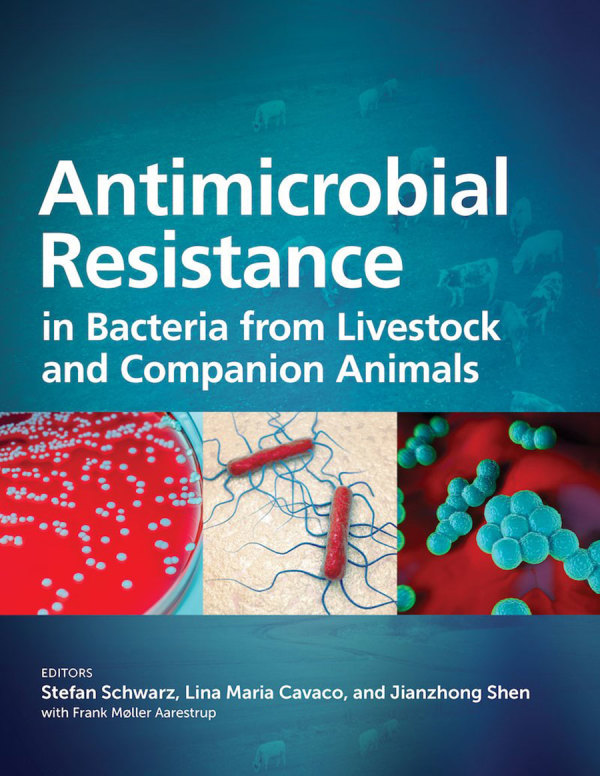
Antimicrobial Resistance in Bacteria from Livestock and Companion Animals

The text begins with a historical overview of the discovery of antimicrobial drugs and a detailed characterization of the indications for and regulation of their use in veterinary medicine. Salient technical issues are discussed, including antimicrobial susceptibility testing in veterinary pathogens, diagnostic methods for detecting antimicrobial resistance, and licensing of antimicrobial drugs. Overviews of the mechanisms of resistance to antimicrobial agents, including antibiotics, metals, and biocides, provide context to the main substance of the text: an exhaustive report of current antimicrobial resistance in a wide range of pathogens of veterinary and medical importance. The text closes with a look into the future of mitigating antimicrobial resistance in veterinary and production settings through monitoring, surveillance, and antimicrobial stewardship.

*Antimicrobial Resistance in Bacteria from Livestock and Companion Animals* presents a wealth of information and is a critical resource for anyone who studies, treats, or is affected by antimicrobial resistance in domesticated animals or the food products that come from them. Contributing authors are globally renowned experts in the field who have composed thoughtful and insightful accounts that generally walk the line between technically thorough and accessible to a broad audience. Whether one is interested in a specific pathogen or in policy to mitigate antimicrobial resistance, this text offers a comprehensive review of the increasingly urgent topic that is antimicrobial resistance in animal-derived pathogens.

